# Quit and Reduction Rates for a Pilot Study of the American Indian Not On Tobacco (N-O-T) Program

**Published:** 2005-09-15

**Authors:** Dr. Kimberly Horn, Geri Dino, Karen Manzo, Lyn McCracken, N Noerachmanto, Tim McGloin, Lynn Lowry-Chavis, Lawrence Shorty

**Affiliations:** Department of Community Medicine, Centers for Public Health Training and Research, Office of Drug Abuse Intervention Studies (ODAIS), West Virginia University; Centers for Public Health Training and Research, ODAIS, West Virginia University, Morgantown, WVa; Centers for Public Health Training and Research, ODAIS, West Virginia University, Morgantown, WVa; Centers for Public Health Training and Research, ODAIS, West Virginia University, Morgantown, WVa; Centers for Public Health Training and Research, ODAIS, West Virginia University, Morgantown, WVa; Center for Health Promotion and Disease Prevention, University of North Carolina–Chapel Hill, Chapel Hill, NC; Center for Health Promotion and Disease Prevention, University of North Carolina–Chapel Hill, Chapel Hill, NC; Center for Health Promotion and Disease Prevention, University of North Carolina–Chapel Hill, Chapel Hill, NC

## Abstract

**Introduction:**

American Indian youths smoke cigarettes at high rates, yet few smoking-cessation programs have been developed for them. The objective of this study, conducted during 2003 and 2004, was to determine the preliminary quit and reduction outcomes of the American Lung Association's newly adapted American Indian Not On Tobacco (N-O-T) program.

**Methods:**

Seventy-four American Indian youths aged 14 to 19 years in North Carolina were enrolled in the American Indian N-O-T program or a brief 15-minute intervention. Quit and reduction rates were compared 3 months after baseline using compliant subsamples and intention-to-treat analyses.

**Results:**

Among males in the American Indian N-O-T program, between 18% (intention-to-treat) and 29% (compliant subsample) quit smoking. Six males (28.6%) in the American Indian N-O-T program reported quitting smoking; one male (14.3%) in the brief intervention reported quitting. No females in either group quit smoking. More females in the American Indian N-O-T program reduced smoking than females in the brief intervention.

**Conclusion:**

These pilot results suggest that the American Indian N-O-T program offers a useful and feasible cessation option for American Indian youths in North Carolina. Program modifications are necessary to improve outcomes for American Indian females, and recruitment issues require in-depth study. Further study is warranted to determine program efficacy.

## Introduction

American Indians are defined by the Surgeon General as "persons who have origins in any of the original peoples of North America and who maintain that cultural identification through self-identification, tribal affiliation, or community recognition" (1). American Indians smoke manufactured cigarettes at rates higher than any other U.S. subgroup, with smoking rates among adults (individuals aged 18 years or older) at nearly 41% (1). Similarly, American Indian youths smoke at rates approaching 50% (2); by seventh grade, 72% of American Indian youths have tried smoking (3). A recent report released by the Substance Abuse Mental Health Services Administration (4) showed that 36% of American Indians aged 12 years or older reported having smoked in the previous month. Unfortunately, cessation strategies for native populations are limited (1,4,5). The American Legacy Foundation's executive summary on priority populations reported that few efforts have been made to develop tobacco-cessation programs for American Indian teenagers. National recommendations to address tobacco use among population groups (*Healthy People 2010* objectives 27.1 and 27.2) (1,6) and federal funding initiatives to reduce health problems among American Indians have focused minimally on tobacco.

In national efforts to promote health and well-being among American Indians, tobacco has been relatively "untouchable." Some experts believe that these failures result from the blurred distinction within the dominant white culture between traditional sacred tobacco use and commercial secular tobacco use and addiction (7). Interestingly, traditional sacred tobacco is seldom grown, and more American Indians are becoming addicted to commercial tobacco — especially cigarettes (8,9). Moreover, American Indians in certain regions of the country trade and grow commercial tobacco. These factors, compounded by increased marketing of tobacco to American Indians, increase the likelihood that they will become addicted to tobacco. What once was a means of spiritual communication now manifests itself more often as a symbol of addiction, disease, disability, and death.

The current study examines the usefulness of the American Lung Association's Not On Tobacco (N-O-T) (9-14) program modified for American Indian teenaged smokers. N-O-T is recognized as a model program of the Substance Abuse and Mental Health Services Administration and as a best practice of the American Lung Association.  N-O-T also received an Innovation in Prevention Research award from the Centers for Disease Control and Prevention's (CDC's) Prevention Research Centers program in 2004. More information about N-O-T is available from www.lungusa.org*.

Prior studies on predominantly white youths have demonstrated that the N-O-T program 1) significantly affects smoking cessation and reduction; 2) is well received by teenagers and facilitators; and 3) benefits participants in areas of their lives other than smoking cessation (e.g., school attendance, stress management, physical activity) (11-13). Despite attempts to reach a diverse youth audience, N-O-T programs have included few American Indians. Moreover, little is known about how to recruit American Indian youths into the N-O-T program and to ensure that the N-O-T program is at least as effective for American Indians as it is for the general population. The present investigation compared the newly adapted American Indian N-O-T program with a brief 15-minute intervention by examining group differences in the 3-month post-baseline quit and reduction rates among American Indian teenaged smokers.

## Methods

### Participants

American Indian smokers aged 14 to 19 years were recruited in North Carolina among high schools in state-recognized tribal areas during early fall semester 2003. The final baseline sample included 74 youths (54 American Indian N-O-T participants and 20 brief intervention participants). Overall, 82.2% of the youths were American Indian, and 60.3% were male. The mean age was 16 years. Youths were included in the study if they were current smokers (i.e., had smoked at least one cigarette in the past 30 days), volunteered to participate, and provided written participant assent and written parental consent. Institutional Review Board (IRB) approval was obtained from West Virginia University and the University of North Carolina. The study adhered to the nondiscriminatory IRB policies and procedures of the two academic institutions and obtained tribal council permission and formal tribal approval, as required. Formal research support was offered by the North Carolina Commission of Indian Affairs, tribal councils, tribal community leaders, and representatives from the program's Community Advisory Board (CAB). N-O-T researchers began working with CAB members approximately 12 months before the program was implemented. The 150-member CAB is composed of community members representing the following state-recognized tribes and urban associations: Coharie, Haliwa-Saponi, Lumbee, Meherrin, Occaneechi Saponi, Sappony, Waccamaw-Siouan, Cumberland County Association for Indian People, Guilford Native American Association, Metrolina Native American Association, and Triangle Native American Society. The CAB provided guidance throughout all study phases.

### Setting

According to data from the North Carolina Commission of Indian Affairs, North Carolina is home to nearly 100,000 American Indians representing eight tribes and four urban associations. The study took place in public high schools in northeastern, southeastern, and central North Carolina counties. The following criteria were used to select schools that were comparable: 1) racial composition (i.e., schools with a higher than average percentage of American Indian youths); 2) community locale (i.e., schools located close to tribal areas); 3) student population size; 4) student–teacher ratio; and 5) economic status of the community or county in which the school was located (e.g., above or below poverty levels, percentage of students receiving free or reduced-price school lunches).

During the 6 months before student recruitment, we selected American Indian N-O-T schools and brief-intervention schools using the following steps. First, based on the described criteria, a CAB subcommittee and researchers selected six N-O-T schools. Second, the same group selected six brief-intervention schools perceived to be most similar to a N-O-T school based on the defined criteria. The total target was 10 schools; we determined 12 potential sites to allow for refusal. Third, school data were collected on each of the criteria, and a numeric matrix was formulated to ensure that perceptions of school matches were accurate. Our past N-O-T research has demonstrated that community involvement in school selection and matching provides accurate matches (15). Matching at the school level increases the likelihood that both schools and youths are similar at baseline. The final step in selection involved contacting school principals to inform them about the study and seek approval for school participation. In many cases, community members made informal contacts with school officials before the officials were contacted by researchers. Researchers made face-to-face visits with most of the principals; 10 sites agreed to participate. Principals made recommendations for potential facilitators to implement the American Indian N-O-T program or the brief intervention. Facilitators were subsequently contacted by researchers. Facilitators received stipends for their efforts ($300 for the American Indian N-O-T program; $100 for the brief intervention).

### Procedure

**Intervention approaches**

The N-O-T core program consists of 10 hour-long sessions that occur once a week on average. The program addresses topics such as understanding reasons for smoking, preparing to quit, understanding nicotine addiction and withdrawal, accessing and maintaining social support, coping with stress, and preventing relapses (14). N-O-T is delivered in same-sex groups of up to 12 teens and is led by a same-sex facilitator. A detailed description of the N-O-T program can be found elsewhere (12,14).

Throughout this pilot study, community-based participatory research strategies were used to guide development (16). For example, suggestions for American Indian N-O-T curriculum revisions were collected from American Indian youth smokers and nonsmokers, American Indian facilitators already trained in N-O-T, and CAB members, including tribal leaders, parents, clergy, and school personnel. Their input was obtained from focus groups, interviews, surveys, and informal discussions, including testimonials and storytelling. A CAB subcommittee and researchers participated in a 2-day meeting to review and approve program modifications. The American Indian N-O-T adaptation, which is essentially a drop-in module for the N-O-T core program, provides 10 newly tailored sessions. Major additions included the following:

- Facts about tobacco-use rates and health consequences among American Indian populations; enhancement of explanations about addiction
- Information about the history of tobacco among American Indians, providing a historical context to the reason American Indians and Alaska Natives have high tobacco-use rates; explanations about how aboriginal botanicals and traditions came to be replaced by nonreligious uses resulting from commercialization and mass manufacturing of tobacco (8)
- Interactive problem-solving methods that incorporate culturally appropriate and diverse learning styles with a range of options for cultural and traditional activities
- Increased emphasis on group identity and cohesion rather than individual efforts
- Increased use of culturally appropriate graphics, tailored print and audio media, and tobacco prevention and cessation materials with cultural themes, particularly reflected in handouts 
- Increased focus on the impact of a teenager's smoking on family and community, such as information on exposure to secondhand smoke, health risks for family members, and promotion of youth advocacy and leadership
- Inclusion of activity options that involve family members.

The American Indian N-O-T program was presented to youths by trained facilitators in the selected schools. Facilitator training was conducted during a 10-hour session by the research team and members of the American Lung Association and included the following: 1) a study protocol overview, 2) certification in the N-O-T core program, and 3) a review of the American Indian N-O-T version.

The brief 15-minute intervention approximated what teenaged smokers might typically receive in a school setting. Although minimal, this type of intervention provided the opportunity to compare youths participating in some sort of intervention rather than no intervention at all. During the brief intervention, mixed-sex groups were gathered for a single, 15-minute classroom session where they received scripted quit-smoking advice and the CDC brochure "I Quit" (available from www.cdc.gov/tobacco/quit/IQuit.pdf). The brief intervention was administered by school personnel who participated in a 2-hour training session with the research team. Training included an overview of the study and instructions on delivering the scripted 15-minute intervention.

**Participant recruitment**

Recruitment guidelines used in previous N-O-T studies were given to American Indian N-O-T and brief-intervention facilitators. A detailed description of recruitment procedures recommended by the N-O-T program can be found elsewhere (17). All recruitment advertisements and posters for the American Indian N-O-T program were tailored for American Indian youth. Consistent with community-based research principles, community members — including youth, research team members, and native artists — helped to design program logos, graphics, themes, and text. Flyers (unusually sized at 12-in by 12-in) were posted throughout the schools. Facilitators and other school personnel also handed out postcard-size flyers to youths. Schools recruited students over 3 to 7 weeks with assistance from school personnel, community members, and research team program managers during early fall semester 2003. CAB members helped to diffuse information about the American Indian N-O-T program through multiple channels such as churches, powwows, tribal council meetings, and youth groups. Researchers and community members also visited schools and set up recruitment tables where they discussed the program with students and teachers and explained the importance of parental consent. The goal was to recruit 20 youths (10 males and 10 females) from each of 10 schools, totaling 200 youths. Youths were not provided material or financial incentives to enroll in the American Indian N-O-T program or the brief intervention.

**Data measurement and collection instruments**

A battery of pencil-and-paper instruments was administered to participants at baseline and follow-up. All data were collected on site by teams of two to four American Indian and non-American Indian researchers. Smoking status was assessed through self-reported number of cigarettes smoked per day at baseline and at 3-month follow-up through a smoking survey form. Participants were asked the question, "Have you smoked on 1 or more days in the past 30 days?" Participants were identified as *reducers* if their percentage reduction in daily smoking was greater than zero from baseline to 3-month follow-up. At baseline, an individual-information form collected demographic information such as age, race, and sex. Also, a smoking-history form documented baseline information on past rates and patterns of smoking, stage of change (i.e., intent to stop smoking), reasons for smoking, motivation to quit, and confidence in quitting. Motivation and confidence were measured on a 5-point scale (1 = none to 5 = very high). The Fagerstrom Tolerance Questionnaire (FTQ), modified for use with youths, was used to measure nicotine dependence at baseline (6,12). Consistent with FTQ scoring, an aggregate score of 0 to 2 indicates very low nicotine dependence; 3 to 4, low dependence; 5, medium dependence; 6 to 7, high dependence; and 8 to 11, very high dependence (12). Consistent with previous studies, the Cronbach α for the FTQ internal consistency of the study sample was .50 (12,13). A CAB subcommittee and researchers met face-to-face to review and approve all study instruments.

### Study design

The study used a quasi-experimental (nonequivalent) pretest–posttest group design. The brief-intervention control group and the American Indian N-O-T intervention group were compared. Community feedback facilitated group assignments, not randomization. Schools were chosen based on criteria previously identified. After schools were assigned as an American Indian N-O-T school or a brief-intervention school, recruitment of study participants began. American Indian N-O-T and brief-intervention programs did not operate simultaneously in any study school. Baseline data were collected during late fall semester 2003; follow-up data were collected during spring semester.

### Data analysis

**Baseline comparisons**

The original recruitment goal for this study was 200 youths and 10 sites (five American Indian N-O-T intervention schools and five brief-intervention schools). We recruited 79 youths in three N-O-T schools and two brief intervention schools. Five of the original 10 schools were unable to recruit enough youths for participation. Baseline data from American Indian N-O-T and brief-intervention participants were compared to determine the similarity of the two samples before intervention. Independent two-tailed *t* tests were used for comparisons. Analyses were performed on eight critical variables that could be associated with smoking or smoking cessation: age, high school grade, age of smoking onset, number of cigarettes smoked on weekdays and weekends, motivation and confidence to quit smoking, and level of nicotine dependence. We controlled for heightened error by applying the Bonferroni adjustment (.05/8), resulting in a significance level of α = .006.

**Determining quit and reduction rates**

A chi-square test was used to compare quit and reduction rates. Consistent with other research on adolescent smoking cessation, quit and reduction rates were computed using individuals rather than schools as units of analyses because the small number of schools (five schools; three N-O-T programs, two brief interventions) limited power and effect sizes. Analyses were performed on the *compliant subsample* (youths who attended the intervention and who were available for follow-up) and the *intention-to-treat sample* (the total sample at baseline, including youths who were not available for follow-up).

To ensure validity of the compliant subsample analyses, baseline factors were used to assess potential attrition biases (10-12). A problem in teen smoking-cessation studies has been loss of participants at follow-up (i.e., participant failure to return for postintervention data collection). Biases would exist if there were systematic differences between participants who provided follow-up data and those who did not, particularly if differences varied by intervention group or factors related to quitting and reduction. An analysis compared baseline data of youths who provided postintervention data (present) with baseline data of those who did not provide postintervention data (absent). This analysis also assessed whether the differences between the present and absent groups varied by treatment (N-O-T program or brief intervention). A 2 × 2 multivariate analysis of variance (MANOVA) with the variables attrition (present or absent) and treatment (N-O-T or brief intervention) was conducted on baseline variables using recommended procedures for handling missing data (18). Neither the attrition nor the attrition × treatment interaction was significant; no subsequent univariate tests were required (Wilks λ = 0.81; *P* = .14), confirming no systematic bias related to attrition.

## Results

### Baseline comparisons

Youths recruited for this study had similar characteristics; the data collected at baseline show nonsignificant differences in seven of eight characteristics (Table 1). The only significant difference measured was level of confidence in the ability to quit smoking (*t*_72_ = 2.77; *P* = .004).

### Quit rates

Quit status was based on a self-report of a minimum of 24-hour abstinence. Quit rates were determined by group (N-O-T or brief intervention), sex, compliant subsample, and intention-to-treat sample (Table 2).

**Quit rates for compliant subsample**

Quit rates for the compliant subsample represent youths who received the intervention and who reported for follow-up. The compliant quit rates assume that youths who did not attend the follow-up session were absent because of reasons unrelated to smoking cessation (e.g., work, relocation). Data show that youths in the N-O-T group had higher quit rates than youths in the brief intervention. Six (28.6%) of N-O-T males quit smoking, compared with one (14.3%) male in the brief intervention. The difference, however, was not significant. No females quit smoking.

**Quit rates for intention-to-treat sample**

The intention-to-treat sample included all youths who were available at baseline, regardless of the amount of intervention they received. The numerator is the number of youths who reported quitting at follow-up; the denominator is the number of youths who were available at baseline. Intention-to-treat analysis assumes that youths who did not attend the follow-up data collection sessions continued to smoke. Almost 18% of N-O-T males quit smoking compared with 10% of males in the brief intervention. No females quit smoking. Differences were not significant. There was, however, a small but meaningful intervention effect size for males (Cohen's *d* = 0.30).

### Reduction rates

Table 3 shows reduction rates by group (N-O-T and brief intervention), sex, compliant subsample, and intention-to-treat sample (Table 3). Data show that among all youths who did not quit smoking, about one quarter reduced weekday use, regardless of intervention group. N-O-T females comprised the greatest percentage of reducers. Although a greater percentage of youths in the brief intervention reduced weekday and weekend smoking compared with youths in the N-O-T program, youths in the N-O-T program reduced smoking by a greater percentage than youths in the brief intervention. Table 4 shows that the weekend percentage reduction was significantly different between N-O-T participants and brief-intervention participants (*t*_10_ = 1.83; *P* = .049).

## Discussion

The original recruitment goal for this study was 200 youths and 10 sites (five American Indian N-O-T sites and five brief-intervention sites). We were able to recruit 79 youths from three American Indian N-O-T schools and two brief-intervention schools. Youth enrollment was thus lower than expected — 39.5% of the initial youth target. Seventy-four of the 79 youths recruited met the selection criteria of current smoking. Approximately 3 months post-baseline, 53.7% (29/54) of N-O-T youths and 70.0% (14/20) of brief-intervention youths were present for follow-up data collection. Overall, 58.1% (43/74) of eligible study youths participated in the follow-up evaluation. Compared with follow-up rates in our other N-O-T studies, this is the lowest rate (19).

Anecdotal feedback from youths, program facilitators, school personnel, and community members suggest numerous reasons for recruitment and retention challenges for the American Indian N-O-T program. The first of these relates to cultural factors. Eastern North Carolina is a tobacco-growing region and many of the American Indian families in the study communities have strong economic ties to tobacco. Also, tobacco has historically been used by American Indians for spiritual and medicinal purposes. These factors may create ambivalence among youth about participating in a tobacco-cessation program. Second, American Indians place a high value on family and community; American Indian N-O-T adaptation recommendations include information on the impact of secondhand smoke on family and community members. Although family values can support positive behavior change, they also can act as attitudinal and motivational barriers when many household members, including elders, smoke and have lenient attitudes toward smoking tobacco (20). Third, research requirements may deter youth recruitment. For example, youths in the American Indian N-O-T program were not permitted to participate without IRB-approved parental consent forms. Youths may not have provided these forms for various reasons: 1) they may simply have forgotten them; 2) they may have been afraid that their parents would be angry if they knew that they smoked; or 3) formal signed assent and consent forms may have reminded youths of research exploitation of American Indians in the past, causing them to choose not to participate. Nonresearch situations for which parental consent is not required may provide a better understanding of youth willingness to join the American Indian N-O-T program. Fourth, privacy may be a concern for youth from small tribes or communities where a stigma is associated with participating in what might be considered a drug-prevention class. Admitting to an addiction problem or seeking outside help may not be acceptable in some communities. Fifth*,* youths may perceive the school climate to be unsupportive of quit efforts when both teachers and youths are permitted to smoke on school grounds. (In North Carolina, the majority of schools are not tobacco-free.) Only 43 of 115 public school districts or units in North Carolina have adopted the state's optional 100% tobacco-free schools policy. No schools in this investigation were in a county with a tobacco-free policy. 

The final recruitment and retention challenge relates to racial factors. In the multiracial counties in North Carolina where the study was conducted, the ratio of American Indian teachers to American Indian students is low. According to community members, lack of American Indian adult role models or contacts in the schools may hinder support and encouragement for American Indian smoking-cessation efforts. This was illustrated in our challenges to recruit American Indian facilitators. Four of eight facilitators were American Indian; four were white. Research supports the importance of involving American Indian people in tobacco-cessation efforts for American Indians (20,21).

Although student recruitment numbers were low, the study was successful at recruiting the target population of American Indian youth smokers. Overall, 82.2% of youths were American Indian. In addition, half of the facilitators were American Indian. More male than female youths participated in the study. Most participants had been smoking for about 5 years. On average, the youths in this study were smoking about 10 cigarettes per day. Interestingly, the FTQ revealed that the youths in this sample had a low–medium dependence on nicotine despite being daily smokers.

Consistent with other N-O-T studies, the percentage of N-O-T males who quit smoking was twice the percentage of brief-intervention males who quit smoking. The difference was not statistically significant; lack of significance likely resulted from small sample size. The effect size was meaningful in terms of intervention impact. Importantly, the quit rates for the males in this study are equal to or higher than male quit rates in other N-O-T studies of homogenous populations of youth. For example, in a recent 5-year review of N-O-T findings, the mean 3-month end-of-program quit rate for males was between 15.1% (intention-to-treat group) and 20.1% (compliant group) (19). Approximately 10% to 14% of brief-intervention males quit smoking in the current study. This rate is slightly higher than spontaneous or care-as-usual rates found in other studies of smoking cessation among teenagers (19).

Two of our study findings were unusual compared with other N-O-T core studies. One was that fewer females than males joined the American Indian N-O-T program. In past N-O-T core studies with predominantly white youth, male recruitment has been more challenging than female recruitment (10,11). Another unusual finding was that no females quit smoking, which has never occurred in a N-O-T study (19). A 5-year review of N-O-T core studies showed an overall quit rate for females between 14.7% (intention-to-treat group) and 18.5% (compliant group)Two of our study findings were unusual compared with other N-O-T core studies. One was that fewer females than males joined the American Indian N-O-T program. In past N-O-T core studies with predominantly white youth, male recruitment has been more challenging than female recruitment (10,11). Another unusual finding was that no females quit smoking, which has never occurred in a N-O-T study (19). A 5-year review of N-O-T core studies showed an overall quit rate for females between 14.7% (intention-to-treat group) and 18.5% (compliant group). Our American Indian N-O-T pilot findings, along with input from CAB members and tribal representatives, suggest that 1) there may be unique aspects of the social and cultural context of smoking and smoking cessation among American Indian females that call for further study; 2) the American Indian N-O-T curriculum needs further adaptation to meet the needs of American Indian females; and 3) facilitator training needs to incorporate additional information on building relationships with females. Our study is not the first smoking-cessation study to find a low rate of success among American Indian females. King et al found that adult women in minority racial and ethnic populations appear to be less responsive to smoking-cessation programs than white women (22). Some research suggests that American Indian females may be less likely than males or females from other racial and ethnic groups to acknowledge the negative health consequences of tobacco use (20,23). It is important to emphasize, however, that 5 of 19 females in our study succeeded in reducing their smoking.

Among youths who did not quit smoking, a greater number of American Indian N-O-T females than brief-intervention females reduced their smoking. American Indian N-O-T youths reduced smoking by a greater amount than brief-intervention youths. Specifically, American Indian N-O-T youths cut back on their smoking by more than half during the week and more than 75% during the weekend.

### Limitations

Using a quasi-experimental design rather than random assignment may have threatened the validity of our results. We concluded that random assignment would be difficult in this early phase of program study because we had yet not formed relationships with the schools. We thus chose a quasi-experimental design. CAB members helped to guide this decision. By following community-based participatory strategies, we established a foundation of trust among schools and communities in recruiting sites and facilitating participation. CAB members believed this was a necessary step for promoting participation. We selected brief-intervention sites and American Indian N-O-T sites based on common characteristics. The two intervention groups had similar baseline characteristics (Table 1), so the threat to validity resulting from participant differences was reduced. Another limitation was the lack of biochemical validation of self-reported smoking status and lack of documentation of days of continuous abstinence from smoking. We were not able to collect these data because of unexpected time constraints. Previous N-O-T studies have found high agreement between self-reports and exhaled carbon-monoxide–validated quit rates (10).

A final limitation of this study is lack of generalizability. This pilot American Indian N-O-T program was implemented among tribes in North Carolina only. Although tribal commonalities may exist across the United States, we cannot assume that a one-size-fits-all approach is appropriate. As we move forward in efficacy testing, tribal involvement from various regions of the United States is critical. 

### Conclusion

To our knowledge, this is the first study on a smoking-cessation program tailored for American Indian youths, and it is the first examination of the American Indian N-O-T program. Lessons learned will improve methods and strategies in subsequent efficacy trials of American Indian N-O-T and general cessation programming for American Indian youths. The general outcomes of the pilot study highlight four key findings: 1) the American Indian N-O-T program served as cessation aid for males and as a reduction aid for females; 2) study youths seemed ready to change their smoking behavior (i.e., more than half of all available youths reduced cigarette use from baseline); 3) recruitment barriers need to be studied and overcome for greater American Indian youth participation in cessation programs; and 4) curriculum adaptation must give greater attention to cultural and contextual issues, especially related to differences between sexes. The current pilot study is the first step toward understanding the usefulness, efficacy, and long-term sustainability of the American Indian N-O-T program. Future research will focus on youth recruitment, gender issues, additional curriculum modifications, and efficacy testing.

## Figures and Tables

**Table 1:** Baseline Survey Results Showing Similarities Between Participants (N = 74) in American Indian Not On Tobacco (N-O-T) Program High Schools and Brief-Intervention High Schools, North Carolina, 2003–2004

	**American Indian ** **N-O-T Program**		**Brief Intervention**		**Overall^a^**	

	**No.**	**Mean (SD)**	**No.**	**Mean (SD)**	**No.**	**Mean (SD)**
Age, y	51	16.2 (1.2)	20	17.1 (1.4)	71	16.5 (1.3)
High school grade (9–12)	52	10.2 (1.2)	19	10.8 (1.3)	71	10.4 (1.2)
Age of smoking onset, y	54	11.6 (2.8)	20	12.2 (3.0)	74	11.8 (2.8)
No. cigarettes smoked per day during weekdays (Monday through Friday)	53	8.8 (12.3)	20	13.2 (10.1)	73	10.0 (11.8)
No. cigarettes smoked per day during weekends (Saturday and Sunday)	52	10.5 (12.1)	19	17.0 (14.4)	71	12.2 (13.0)
Motivation to quit^b^	54	3.0 (0.9)	20	2.8 (1.1)	74	2.9 (1.0)
Confidence in ability to quit^b^ (*P* = .004)	54	3.1 (1.0)	20	2.4 (0.9)	74	2.9 (1.1)
Dependence on nicotine^c^	51	4.2 (1.4)	17	4.5 (1.7)	68	4.3 (1.5)

a When combined data do not equal 74, data are missing.

b These items were measured on a Likert scale of 1–5 with 1 indicating no motivation (or confidence) and 5 indicating very high motivation (or confidence).

c The Fagerstrom Tolerance Questionnaire (FTQ) modified for use with youth was used to measure nicotine dependence at baseline. A score of 0–2 indicates very low nicotine dependence; 3–4, low dependence; 5, medium dependence; 6–7, high dependence; 8–11, very high dependence (12).

**Table 2:** Survey Results 3 Months After Baseline Comparing Quit Rates Among Participants in American Indian Not On Tobacco (N-O-T) Program and Brief Intervention, North Carolina, 2003–2004

	**American Indian ** **N-O-T Program**		**Brief Intervention**		**Overall**	

	**No. Parti-** **cipants**	**No. Who Quit (%)**	**No. Parti-** **cipants**	**No. Who Quit (%)**	**No. Parti-** **cipants**	**No. Who Quit (%)**

**Compliant Subsample^a^**						

Male	21	6 (28.6)	7	1 (14.3)	28	7 (25.0)
Female	8	0 (0.0)	7	0 (0.0)	15	0 (0.0)
Total	29	6 (20.7)	14	1 (7.1)	43	7 (16.3)

**Intention-to-Treat Subsample^b^**						

Male	34	6 (17.6)	10	1 (10.0)	44	7 (15.9)
Female	20	0 (0.0)	10	0 (0.0)	30	0 (0.0)
Total	54	6 (11.1)	20	1 (5.0)	74	7 (9.5)

a The compliant subsample included youths who attended the intervention and who were available for follow-up.

b The intention-to-treat subsample included the total sample at baseline, including youths who were not available for follow-up.

**Table 3:** Smoking Reduction Rates Among Participants in American Indian Not On Tobacco (N-O-T) Program and Brief Intervention, North Carolina, 2003–2004

	**American Indian ** **N-O-T Program**		**Brief Intervention**		**Overall **	

	**No. Parti-** **cipants**	**No. Who ** **Reduced (%)**	**No. Parti-** **cipants**	**No. Who ** **Reduced (%)**	**No. Parti-** **cipants**	**No. Who ** **Reduced (%)**

**Compliant Subsample^a^**						

**Weekdays**						

Male	15	6 (40.0)	6	3 (50.0)	21	9 (42.9)
Female	8	5 (62.5)	7	2 (28.6)	15	7 (46.7)
Total	23	11 (47.8)	13	5 (38.5)	36	16 (44.4)

**Weekends**						

Male	15	4 (26.7)	6	3 (50.0)	21	7 (33.3)
Female	8	2 (25.0)	7	3 (42.9)	15	5 (33.3)
Total	23	6 (26.1)	13	6 (46.2)	36	12 (33.3)

**Intention-to-Treat Subsample** b						

**Weekdays**						

Male	28	6 (21.4)	9	3 (33.3)	37	9 (24.3)
Female	20	5 (20.0)	10	2 (20.0)	30	7 (23.3)
Total	48	11 (22.9)	19	5 (26.3)	67	16 (23.9)

**Weekends**						

Male	28	4 (14.3)	9	3 (33.3)	37	7 (18.9)
Female	20	2 (10.0)	10	3 (30.0)	30	5 (16.7)
Total	48	6 (12.5)	19	6 (31.6)	67	12 (17.9)

a The compliant subsample included youths who attended the intervention and who were available for follow-up.

b The intention-to-treat subsample included the total sample at baseline, including youths who were not available for follow-up.

**Table 4:** Mean Percentage Change in Amount of Smoking Reported by Participants in American Indian Not On Tobacco (N-O-T) Program and Brief Intervention, North Carolina, 2003–2004

	**American Indian N-O-T Program**		**Brief Intervention**		**Overall**		

	**No. Who Reduced**	**Change (SD)**	**No. Who Reduced**	**Change (SD)**	**No. Who Reduced**	**Change (SD)**	***P* value**
Weekdays	11	−58.4% (28.5%)	5	−55.4% (23.3%)	16	−57.5% (28.5%)	.84
Weekends	6	−78.2% (28.3%)	6	−49.6% (25.8%)	12	−63.9% (29.8%)	.049
